# Orthoflavivirus infection and the mTOR signaling pathway

**DOI:** 10.3389/fmicb.2025.1565350

**Published:** 2025-04-09

**Authors:** Yahui Zhang, Jianbo Ba, Jie Luan, Zhongtian Qi, Bin Liu

**Affiliations:** ^1^Naval Medical Center, Naval Medical University, Shanghai, China; ^2^Department of Cardiology, Shanghai East Hospital, Tongji University, Shanghai, China; ^3^Department of Microbiology, Naval Medical University, Shanghai, China

**Keywords:** orthoflavivirus, dengue virus, Zika virus, mTOR signaling pathway, viral replication

## Abstract

Each year, mosquito-borne orthoflaviviruses, including Zika virus, dengue virus, and the Japanese encephalitis virus, threaten the health of more than 400 million people worldwide. To date, knowledge about the pathogenic mechanisms underlying orthoflavivirus infection and the interactions of these viruses with host cells is limited. Mammalian target of rapamycin (mTOR) is pivotal for cell growth and metabolism. The downstream targets of mTOR regulate protein translation and cell autophagy to affect orthoflavivirus replication, and its upstream protein AKT performs similar functions. In this work, the mechanism underlying the relationship between the mTOR signaling pathway and orthoflavivirus infection was reviewed from three perspectives: orthoflavivirus structure and life cycle, mTOR structure and signaling pathway, and regulation of the mTOR signaling pathway during orthoflavivirus infection.

## 1 Introduction

Orthoflaviviruses, including Zika virus (ZIKV), dengue virus (DENV), Japanese encephalitis virus (JEV), West Nile virus (WNV), and yellow fever virus (YFV), are predominantly transmitted by mosquitoes, putting humans at risk, especially in tropical and subtropical areas. In 2015, the reemergence of a ZIKV outbreak in America was associated with Guillain-Barré syndrome and microcephaly, and this outbreak was declared by the World Health Organization (WHO) to be a public health emergency of international concern (PHEIC) from February to November 2016 (Petersen et al., 2016). In addition, the incidence of dengue cases has grown dramatically worldwide in recent decades. In America, over 4.5 million dengue cases, resulting in 2300 deaths, were reported by the WHO, and DENV spread to over 100 countries in 2023 due to the changing distribution of *Aedes* vectors (Pourzangiabadi et al., 2024). The epidemics caused by orthoflaviviruses pose constant threats to human health; unfortunately, no effective vaccines or drugs are available owing to the limited knowledge of the pathogenesis underlying orthoflavivirus infection and the interactions of these viruses with host cells (Bello et al., 2024). Mechanistic target of rapamycin (mTOR), an important host protein involved in coordinating eukaryotic cell growth and metabolism, has been shown to participate in viral replication. Evolutionarily conserved mTOR, an atypical serine/threonine protein kinase, belongs to the PI3K-related kinase family and acts as a catalytic subunit of two complexes, namely mTOR complex 1 (mTORC1) and 2 (mTORC2). The mTOR signaling pathway integrates various extracellular signals, including stress, energy, and growth factors, and participates in various biological processes (Saxton and Sabatini, 2017; Panwar et al., 2023). This work summarizes the correlation between the mTOR signaling pathway and orthoflavivirus replication, which may provide new insight into the development of drugs and vaccines to treat or prevent orthoflavivirus infection.

## 2 Structure and life cycle of orthoflaviviruses

**Figure 1:**
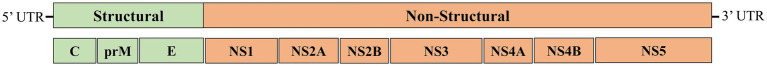
The schematic of orthoflavivirus genome organization.

**Figure 2:**
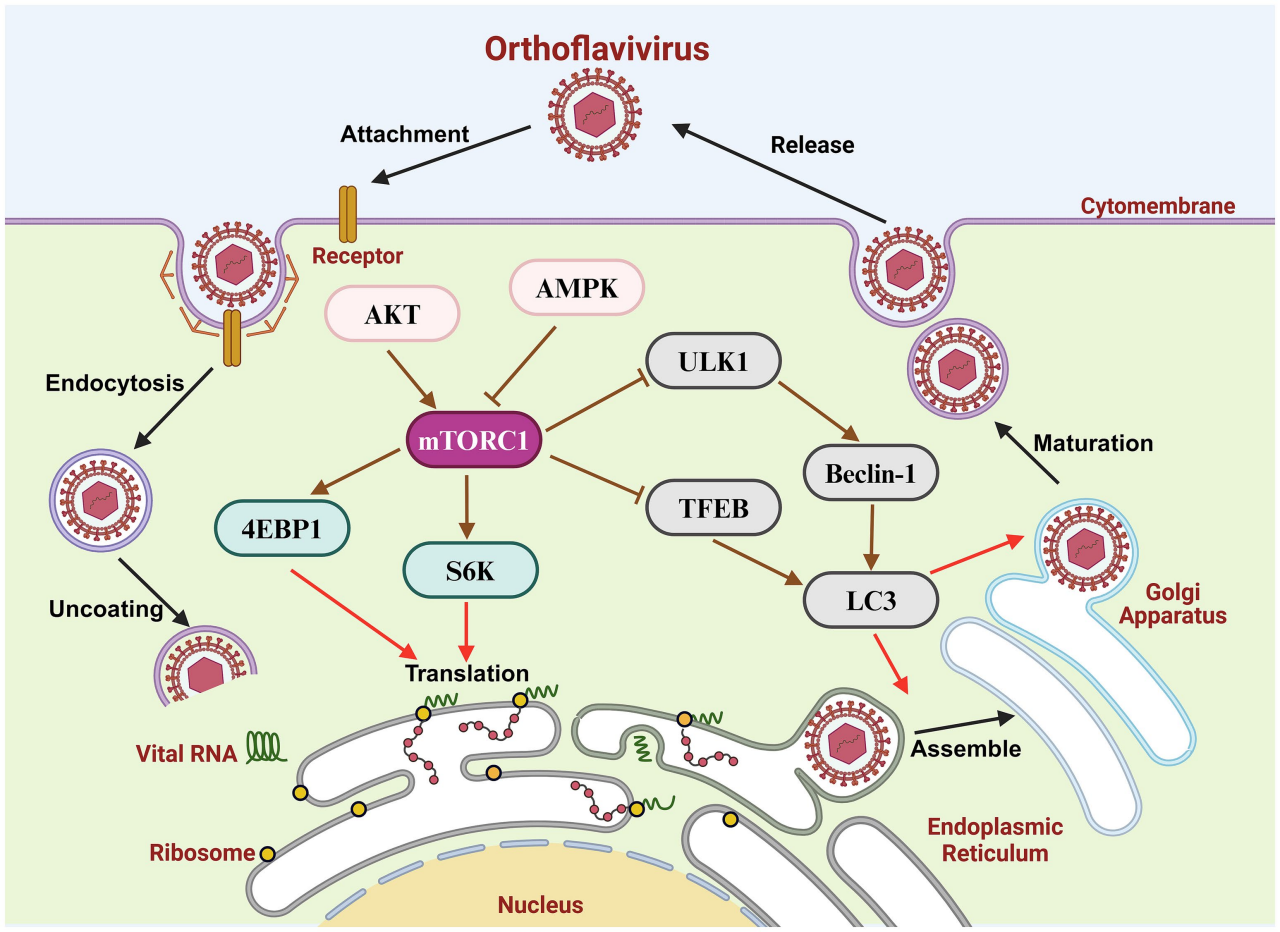
Orthoflavivirus replication cycle and mTOR signaling pathway.

Orthoflaviviruses, which belong to the family *Flaviviridae*, are single-stranded, positive-sense RNA viruses approximately 50 nm in diameter. The genomes of orthoflaviviruses are approximately 11 kb in length and encode a polyprotein, which is processed into three structural proteins [precursor membrane protein (prM), envelope protein (E), and capsid protein (C)] and seven non-structural proteins (NS1, NS2A, NS2B, NS3, NS4A, NS4B and NS5) (Figure 1; Lindenbach and Rice, 2003). Virions bind to cell surface receptors through the E protein and enter the cytoplasm through receptor-mediated endocytosis. Membrane fusion of virions and endosomes leads to virion uncoating and the release of viral genomes. The virion RNA is translated into a polyprotein, and subsequent cleavage is catalyzed by intracellular or NS2B/NS3 proteases. The N-terminus of NS3 contains a serine protease domain that binds to the cofactor NS2B to form the NS2B/NS3 protease in the endoplasmic reticulum (ER), which functions in the cleavage of viral polymerases. The virion RNA is amplified depended on NS5; the C-terminal region of NS5 is an RNA-dependent RNA polymerase (RdRp), and an N-terminal region is a methylase (MTase). The progeny RNA strands are then separated from the template strands by the C-terminus of NS3, which has RNA helicase activity and nucleoside triphosphatase activity. The C protein encloses the viral genome to form a nucleocapsid and assembles with the E protein and prM protein on the ER. Immature virions are transmitted to the Golgi apparatus, resulting in the cleavage of prM into the mature M protein by furin protease. Mature virions are then transported to the plasma membrane and exosomes may play a role in the viral release (Figure 2; Kaufmann and Rossmann, 2011; Morita and Suzuki, 2021; Singh et al., 2025).

## 3 mTOR structure and signaling pathway

mTOR forms two complexes with different functions and regulatory mechanisms. mTORC1 is composed of three core components: mTOR, Raptor and mLST8. Raptor binds the TOR signaling motif to facilitate substrate recruitment to mTORC1 and guarantee its accurate subcellular localization. mLST8 binds the catalytic domain of mTORC1 and stabilizes its high catalytic activity. mTORC1 is sensitive to inhibition by rapamycin, which binds to its FKBP12 domain to inhibit its downstream signaling for cell growth and proliferation. In contrast, mTORC2 is insensitive to rapamycin and consists of mTOR, mLST8 and Rictor rather than Raptor. mTORC1 activity shifts in response to extracellular stimuli, such as stress, growth factors, or energy changes. Activation of the mTORC1 signaling pathway can inhibit autophagy, but it promotes synthesis of nucleotides, proteins and lipids to increase cell growth and proliferation. mTORC2 is predominantly modulated by growth factors to regulate cell proliferation and survival (Saxton and Sabatini, 2017).

mTORC1 can promote protein translation mainly through the dephosphorylation of its downstream targets, eIF4E binding protein (4E-BP) and p70 ribosomal protein S6 kinase (S6K) (Nojima et al., 2003). In addition, mTORC1 can also downregulate the activity of PKR-like endoplasmic reticulum resident kinase indirectly and then dephosphorylate eIF2α to decrease transcription factor 4 expression, resulting in unfolded protein response and disturbing protein translation (Li et al., 2023). Autophagy activity is inhibited by the mTORC1 signaling pathway under conditions of adequate nutrition. mTORC1 phosphorylates the S757 site of UNC-51-like kinase 1 (ULK1) to interfere with autophagosome formation and ULK1 activation via AMPK, inhibiting autophagy (Kim et al., 2011). Transcription factor EB (TFEB), another downstream target of mTORC1, also inhibits autophagy. TFEB is phosphorylated by mTORC1 and hinders its nuclear translocation, as well as the expression of genes related to lysosomal biogenesis (Martina et al., 2012). mTORC2 can phosphorylate AKT and activate the PI3K/AKT signaling pathway to regulate cell metabolism. AKT activation also enhances mTORC2 activity, in turn, by acting as a positive feedback mechanism (Sarbassov et al., 2005). With respect to upstream factors of mTORC1, the tuberous sclerosis complex (TSC), which is regulated by several upstream factors, such as AKT, MAPK, and AMPK, inhibits brain-enriched Ras homologs to depress mTORC1 activity. Importantly, there is an antagonistic effect on TSC2 phosphorylation between AMPK and AKT (Long et al., 2005).

## 4 The mTOR signaling pathway regulates orthoflavivirus replication

Numerous studies have revealed that the mTORC1 signaling pathway is highly involved in the infection and replication of many viruses. On the one hand, some viruses activate the mTORC1 signaling pathway to benefit their replication. Rotavirus infection activates the PI3K/AKT/mTORC1 signaling pathway to inhibit autophagy, promoting viral self-replication (Yin et al., 2018), and the influenza A virus protein NS1 phosphorylates AKT to inhibit REDD1 activity and activate mTORC1 promote viral replication in the later stage of infection (Kuss-Duerkop et al., 2017). On the other hand, mTORC1 activity is inhibited during infection by some viruses. The F17 protein of poxvirus inhibits the mitochondrial mTORC1 signaling pathway to hinder the interferon-stimulated gene response but promotes the synthesis of viral proteins (Meade et al., 2023), and the ORF7a protein of SARS-CoV-2 initiates autophagy and limits autophagosome-lysosome fusion by inhibiting the mTORC1/ULK1 signaling pathway, thereby promoting progeny virus production (Hou et al., 2023). The mTOR signaling pathway has also been proven to play an important role in orthoflavivirus infection and replication (Table 1).

**Table 1:** The related references of mTOR signaling pathway regulated by orthoflavivirus infections.

Orthoflavivirus	mTORC1	mTORC1 upstream		mTORC1 function		References
		AKT	AMPK	Protein translation	Autophagy	
ZIKV	+	+		+		Lin et al. (2019), Sahoo et al. (2020), Albentosa-González et al. (2021a), Viettri et al. (2023), and Palmero Casanova et al. (2024)
	−	−		−	+	Liang et al. (2016), Wang et al. (2021), Liu et al. (2023), and Leite-Aguiar et al. (2024)
	−		+		+	Qin et al. (2022)
DENV	−			−	+	Kao et al. (2018), Kong et al. (2020), Wang et al. (2021), Carter et al. (2022), Sinha et al. (2022), and Zheng et al. (2022)
	−		+	−	+	Jordan and Randall (2017), Wu et al. (2022), and Wu et al. (2023)
	−	−		−	+	Lahon et al. (2021)
JEV	−	−			+	Huang et al. (2016), Prajapat et al. (2024), and Singh et al. (2024)
WNV	+	+		+		Shives et al. (2014) and Shives et al. (2016)
USUV	+	+				Albentosa-González et al. (2021b)

+ Activation; − Inactivation.

### 4.1 mTOR signaling regulates protein translation to influence orthoflavivirus replication

The mTOR signaling pathway modulates the synthesis of orthoflavivirus proteins via the phosphorylation of S6K and 4E-BP. mTORC1 directly phosphorylates T389 of S6K, which is a key site in the hydrophobic domain, followed by phosphorylation and activation of S6K by pyruvate dehydrogenase kinase 1, thereby activating eukaryotic translation initiation-related factors. S6K also mediates the degradation of programmed cell death factor 4 (PDCD4) to increase the activity of eukaryotic translation initiation factor (eIF) 4B and promote the translation of mature mRNA (Holz et al., 2021). Unlike S6K, 4E-BP binds to and isolates eIF4E to prevent the assembly of the eIF4F complex and inhibit protein synthesis. mTORC1 phosphorylates multiple sites of 4E-BP to dissociate it from eIF4E and initiate 5-terminal-dependent mRNA translation (Gingras et al., 1999).

Studies have largely demonstrated that the mTOR signaling pathway is inhibited during ZIKV and DENV infection and that viral protein translation is hindered. S6K phosphorylation and mTORC1 activity are reduced in DENV-infected cells, including human umbilical vein endothelial cells (HUVECs), HepG2 cells, and MEG-01 cells (Jordan and Randall, 2017; Lahon et al., 2021; Wang et al., 2021; Carter et al., 2022; Sinha et al., 2022; Wu et al., 2022; Wu et al., 2023). In contrast, during DENV infection in HUVEC and HepG2 cells, mTORC1/S6K signaling is inhibited by the activation of upstream AMPK (Wu et al., 2022; Wu et al., 2023), whereas in MEG-01 cells, mTORC1/S6K signaling is inhibited by AKT inactivation (Lahon et al., 2021).

The protein translation activity of mTORC1 in different cells during ZIKV infection remains controversial. Leite-Aguiar et al. (2024) reported that ZIKV infection inhibits the mTOR signaling pathway in the brain tissue of C57BL/6 mice, dephosphorylates S6K, and phosphorylates 4E-BP to block protein translation. ZIKV infection exacerbates neurological damage and inflammation, but the viral load in brain tissue is decreased. Similarly, our previous study revealed that ZIKV-infected HUVECs inhibit the mTOR signaling pathway to block viral protein translation through S6K and 4E-BP (Liu et al., 2023). These results indicate that mTOR signaling pathway activation may be required for viral protein synthesis during virus replication. In contrast, Mercedes Viettri et al. reported that the mTORC1 signaling pathway is activated in ZIKV-infected trophoblastic cells or lymphoblastoma cells and that S6K is phosphorylated to promote viral protein replication (Viettri et al., 2023). Similarly, Sahoo et al. (2020) reported that ZIKV infection activates mTORC1 and S6K in neuronal precursor cells and glial cells. The regulatory action of the mTORC1 signaling pathway in different cells during ZIKV infection differs, but the aforementioned findings emphasize the importance of mTORC1 activation in protein translation during ZIKV replication. Viral protein synthesis requires the function of mTORC1 through the activation of its downstream targets S6K or 4E-BP. However, the differing activities of the mTORC1 signaling pathway may be due to the comprehensive effects of the host response and ZIKV infection. The protein translation function mediated by mTORC1 signaling is suppressed in endothelial cells, whereas it is enhanced in tumor cells and neural cells, resulting from expression variability of intracellular protein across distinct cell types. Furthermore, mTORC1 signaling is differentially regulated in ZIKV-infected brain tissues or neural cells because of different hierarchies *in vitro* versus *in vivo*. Additionally, post-infection time or metabolic stress may also attribute to the mTORC1-regulated protein expression during ZIKV infection and the precise mechanisms underlying these differential regulatory effects warrant further investigation.

Surprisingly, WNV infection activates AKT/mTORC1/S6K signaling to promote viral growth and viral protein expression, and Raptor, which is the core component of TORC1, also shows increased expression, which has a limited effect on genome replication (Shives et al., 2014). In addition, there are no reported studies on the role of the mTOR signaling pathway in protein translation during JEV and YFV infection.

### 4.2 Autophagy is induced during orthoflavivirus infection via mTOR signaling

Autophagy is a dynamic, highly conserved catabolic process. During autophagy, the intracellular membrane is rearranged to engulf long-lived cytoplasmic components or damaged organelles, forming double-membraned autophagosomes. Mature autophagosomes ultimately fuse with lysosomes to form single-membrane autophagolysosomes to allow the degradation or recycling of their contents (Klionsky and Emr, 2000). Microtubule-associated protein 1 light chain 3 (LC3) is converted from LC3-I to the lipidated LC3-II and anchored to the autophagic membrane upon autophagy initiation. LC3-II distribution represents the autophagosomes accumulation and regard as a marker of autophagy induction. Moreover, p62, degraded by the autophagic-lysosome pathway, usually interacts with LC3-II to reflect the occurrence of complete autophagic flux (Mizushima, 2025). mTORC1 regulates autophagy by phosphorylating ULK1 and TFEB. ULK1 phosphorylates Beclin-1 at the Ser14 site, thereby enhancing the activity of the VPS34 complex composed of ATG13, FIP200, and ATG101. It promotes the recruitment of autophagy-related proteins to autophagosomal membranes and autophagy initiation. However, mTORC1-mediated phosphorylation of ULK1 at Ser637 and Ser757 inhibits this process (Kim et al., 2011). TFEB can be phosphorylated and inhibited by mTORC1 to hinder the expression of genes for lysosomal biogenesis related to the autophagy machinery. The rearranged membrane structures on autophagy induction are utilized as a stable compartment for genome replication and virion maturation in orthoflaviviruses, and the degraded or recycled contents can then be used as raw material for viral replication (Song et al., 2024). Researches have proved that autophagy is induced during orthoflavivirus infection via the AKT/mTORC1/ULK1/Beclin-1 signaling pathway. DENV infection in HUVECs, HepG2 cells, MEG-01 cells, and A549 cells induces autophagy and the conversion of LC3-I to lipidated LC3-II (Jordan and Randall, 2017; Kao et al., 2018; Lahon et al., 2021; Wang et al., 2021; Carter et al., 2022; Wu et al., 2023). Autophagy induction by DENV infection may require the activation of AMPK. The DENV NS1 protein promotes the AMPK-LKB1 interaction to activate the AMPK/ERK/mTORC1 signaling pathway, thereby inducing autophagy in HUVECs (Wu et al., 2022; Wu et al., 2023). However, Lahon et al. (2021) reported that DENV infection in MEG-01 cells suppresses AKT activation to inhibit the mTORC1 signaling pathway and induce autophagy, which inhibits the development and maturation of megakaryocytes.

Given the severe neurological symptoms of ZIKV infection, the regulatory mechanism in the nervous system is of great concern. Liang et al. (2016) reported that in human fetal neural stem cells (fNSCs), the ZIKV proteins NS4A and NS4B inhibit the AKT/mTORC1/ULK1/Beclin-1 signaling pathway, causing defective neurogenesis and aberrant autophagy activation. However, Bikash RSahoo et al. reported that ZIKV infection in human neuronal precursors and glial cells transiently induces autophagy in early infection stages by inhibiting mTORC1 activity to counter virus replication. However, both mTORC1 and mTORC2 are subsequently activated in later stages of ZIKV infection, and autophagy is negatively regulated through the mTORC1/ULK1 signaling pathway, thereby facilitating ZIKV replication. Torin l or rapamycin, mTORC1 inhibitors, significantly reduce viral protein expression and progeny production Sahoo et al. (2020). Contrasting conclusions suggest that more studies are needed to further explore the correlation between ZIKV infection and autophagy in nerve cells and tissues. Our previous study revealed that autophagy is induced via the mTORC1/ULK1/Beclin-1 signaling pathway in ZIKV-infected HUVECs to provide a compartment for viral replication and that mTORC1 inactivity simultaneously blocks viral protein expression, with the former exerting a stronger effect than the latter on viral replication. Similarly, Wang et al. (2021) reported that ZIKV-infected HUVECs induce autophagy to promote viral replication through the mTORC1/TFEB signaling pathway. Additionally, the lipophagy, which is a type of selective autophagy, occurs in ZIKV-infected Huh 7 cells through the mTORC1/ULK1 pathway to increase viral replication (Qin et al., 2022). Moreover, the role of mTOR signaling pathway-regulated autophagy in ZIKV replication has been verified clinically. Carlos Fernando et al. sequenced blood samples from ZIKV-infected patients and reported that the PI3K/AKT/mTORC1 pathway was inhibited and that multiple autophagy-related genes were upregulated (Melo et al., 2017). These results are consistent with the conclusions of Sahoo et al., indicating that ZIKV infection induces autophagy to promote viral replication. In addition, mTORC2 can activate AKT and further regulate autophagy or protein translation to influence ZIKV replication (Viettri et al., 2023). Several studies have also indicated that JEV infection inhibits mTORC1 activity to induce autophagy and increase viral replication (Prajapat et al., 2024; Singh et al., 2024). Interestingly, WNV growth and progeny production are independent of autophagy activation (Beatman et al., 2012).

## 5 The development of mTOR signaling-targeting drugs against orthoflaviviruses

Drugs targeting the mTOR signaling pathway have been explored as potential inhibitors of orthoflavivirus infections. Inhibition of AKT, an upstream regulator of mTORC1, demonstrates strong antiviral activity against ZIKV, DENV, and USUV, as seen with compounds like MK-2206, honokiol, and ipatasertib (Albentosa-González et al., 2021a). Notably, the non-competitive inhibitor miransertib exhibits greater efficacy in suppressing ZIKV and USUV replication compared to the competitive inhibitor capivasertib (Palmero Casanova et al., 2024). Additionally, compound C, which inactivates AMPK of another mTORC1 upstream modulator, reduces DENV replication and progeny virion production (Jordan and Randall, 2017). Direct mTORC1 inhibition—using rapamycin, Torin 1, AZD8055, or KU0063794—also restricts DENV, ZIKV, WNV, and YFV infections (Shives et al., 2014; Lahon et al., 2021; Viettri et al., 2023). However, some studies reported contradictory effects; for instance, rapamycin and its analogs enhance ZIKV replication in epithelial and neural cells (Liang et al., 2016; Liu et al., 2023) and inhibition of mTOR by rapamycin suppressed antiviral and anti-inflammatory gene expression during YFV infection (Cao et al., 2008). Promisingly, several novel mTOR-targeting compounds show potent anti-orthoflavivirus activity. Cyclovirobuxine D enhances mTORC1 activity and blocks viral-dependent autophagy, thereby inhibiting ZIKV and DENV replication (Wang et al., 2021). Quercetin from *E. perfoliatum* extract binds with higher affinity to the DENV receptor TIM-1, exerting antiviral effects against DENV infection (Lahon et al., 2021). Phloretin reduces viral production by suppressing AKT/mTORC1 phosphorylation (Lin et al., 2019). CW-33, an intermediate synthesized derivative of furoquinolines, mitigates JEV-induced cytopathic effects and apoptosis by upregulating AKT/mTOR-associated enzyme modulators (Huang et al., 2016). Cyclosporine A disturbs the NS4B-cyclophilin A interaction by targeting mTOR signaling pathway potentially to inhibit YFV replication (Vidotto et al., 2017). These findings highlight the potential of mTOR pathway inhibitors as broad-spectrum antiviral candidates against orthoflaviviruses.

## 6 Conclusion

Many studies have confirmed that the mTOR signaling pathway plays an essential role in orthoflavivirus infection. mTORC1 affects the synthesis of viral proteins by regulating the phosphorylation of its downstream targets S6K and 4E-BP. Moreover, autophagy is modulated through the mTORC1/ULK1 signaling pathway during orthoflavivirus infection to provide a platform for viral RNA replication and virion maturation (Figure 2). Additionally, AKT, the upstream molecule of mTORC1, is of paramount importance in orthoflavivirus replication. Many studies have shown that DENV or ZIKV infections regulate the AKT/mTORC1 signaling pathway to influence viral replication (Lin et al., 2019; Sinha et al., 2022). The RdRp domain of DENV, ZIKV and USUV NS5 can interact with AKT and activate it to promote virus infection (Albentosa-González et al., 2021a; Albentosa-González et al., 2021b; Carter et al., 2022). Although the regulation of the mTOR signaling pathway in different orthoflavivirus-infected cells varies, changes in this pathway tend to favor viral replication, indicating that the functions of orthoflaviviruses in the mTOR signaling pathway are egoistic. Some drugs, such as rapamycin, capivasertib, and bafilomycin, disrupt the activity of the mTOR signaling pathway and effectively inhibit viral infection and replication, suggesting that mTOR-related molecules are potential targets for anti-orthoflavivirus drug development. Therefore, a better understanding of the interaction between the mTOR signaling pathway and orthoflavivirus infection may provide an important theoretical basis for the prevention and clinical treatment of viral infection.
